# Is the platelet to lymphocyte ratio a promising biomarker to distinguish acute appendicitis? Evidence from a systematic review with meta-analysis

**DOI:** 10.1371/journal.pone.0233470

**Published:** 2020-05-22

**Authors:** Lianjie Liu, Zhuo Shao, Hang Yu, Wei Zhang, Hao Wang, Zubing Mei

**Affiliations:** 1 Department of Colorectal Surgery, Changhai Hospital, Navy Medical University, Shanghai, China; 2 Department of General Surgery, Changhai Hospital, Navy Medical University, Shanghai, China; 3 Emergency Department, Changhai Hospital, Navy Medical University, Shanghai, China; 4 Department of Anorectal Surgery, Shuguang Hospital, Shanghai University of Traditional Chinese Medicine, Shanghai, China; 5 Anorectal Disease Institute of Shuguang Hospital, Shanghai, China; The Ohio State University, UNITED STATES

## Abstract

**Background:**

Although several previous studies have examined the association between the platelet to lymphocyte ratio (PLR) and acute appendicitis (AA), findings have been controversial. We aimed to systematically assess the available evidence to elucidate the overall relationship between the PLR and AA.

**Methods:**

Pubmed and Embase databases were searched for all available published literature before August, 2019 b**y** two independent investigators for observational studies reporting the association between the PLR and AA. Random effects models were applied for all meta-analyses. Pooled standardized mean difference (SMD) and 95% confidence interval (CI) were calculated as effect estimates.

**Results:**

Eleven articles met the inclusion criteria and included in this study. Meta-analysis showed that the level of PLR in the AA group was significantly higher than that in the control group (SMD: 1.19, 95% CI: 0.75 to 1.62, P<0.001). A series of subgroup analyses were conducted to investigate the heterogeneity, showing a significant increase in PLV levels in adults with age ≥30 years (SMD: 1.46, 95% CI: 0.89 to 2.02),compared to those in adult <30 years(SMD: 0.58, 95% CI: 0.12 to 1.04) or in children (SMD: 1.03, 95% CI: 0.51 to 1.56). Compared to non-AA controls, a significant increased PLR level was also observed in non-perforated AA (SMD: 1.23, 95% CI: 0.88 to 1.59) and in AA patients during pregnancy (SMD: 0.70, 95% CI: 0.36 to 1.04), while not in perforated AA (SMD: 2.28, 95% CI: -1.72 to 6.28).

**Conclusions:**

A significant increase in PLR level is found in patients with AA, indicating that PLR is a promising biomarker for AA. PLR provides a convenient option for emergency department to quickly screen for clinically or radiologically confirmed AA awaiting appendectomy, especially for pregnant women suspected of having AA. More high-quality evidence is needed to further confirm the diagnostic accuracy of PLR for AA.

## Introduction

Acute appendicitis (AA) affects approximately 50000 and 300000 individuals annually who receive appendectomies in the UK and in the US, respectively [1]. It has been proposed that AA is characterized by a series of pathophysiological events including obstruction of the appendix lumen, reduced blood flow to the appendix, destruction of mucosal barrier function, bacterial invasion, inflammatory cell infiltration, tissue hypoxia, necrosis and even perforation [2, 3]. It is reported that perforation may occur in 13–20% of AA patients [4, 5].

Due to the uncertainty of its aetiology, the confirmation or elimination of the diagnosis of appendicitis is the primary concern. If AA is suspected, how to stratify simple and complex appendicitis is also highly significant. Though several diagnostic approaches have been widely applied in clinical practice [6–10], the optimum strategy with non-invasiveness or radiation-free imaging modalities or other laboratory examinations has still not reached consensus, representing a long march for both the patients and surgeons to go.

In the last decades, findings from studies on commonly used blood biomarkers are used to aid the diagnosis of suspected AA patient, especially in children, pregnant women or women of fertile age, and elderly patients. Those inflammatory markers, such as C-reactive protein (CRP), mean platelet volume (MPV), platelet (PLT), platelet distribution width (PDW) and red blood cell distribution width (RDW) or other blood biomarkers, can help identify AA with a certain specificity and sensitivity [11–19].

Recent, increasing evidence suggests that platelet to lymphocyte ratio (PLR) may serve as an inflammatory marker used as a diagnostic or prognostic indicator for various diseases [20–25]. PLR has been shown to be a promising predictive factor for patients with suspected AA [26–28]. A recent study also indicated that the PLR was potent predictor for the differential diagnosis of AA and other disease status [29]. However, the clinical significance of this parameter in patients suspected AA remains unclear. The primary aim of the study was to investigate the relationships between the PLR and AA based on the cumulating evidence of published literature.

## Methods

This study was performed and reported according to the Preferred Reporting Items for Systematic Reviews and Meta-analyses (PRISMA) statement [30] and AMSTAR (Assessing the methodological quality of systematic reviews) Guidelines. The protocol for this meta-analysis was registered on PROSPERO (CRD42019146140). The PRISMA checklist is presented in **S1 Table**.

### Literature search

In August, 2019, a comprehensive literature search was performed using online databases b**y** two independent authors, including PubMed, Embase and Cochrane Library. Relevant observational studies were identified that evaluated the association between the PLR level and AA. Search terms including Mesh terms or free text words were listed as follows: “Appendicitis”, “Appendectomy”, “Lymphocyte Count”, “platelet to lymphocyte ratio”, “platelet lymphocyte ratio”, OR “platelet-to-lymphocyte ratio” or “PLR”. Detailed search strategies are provided in **S1 File**. In addition, manual reference search of eligible literature was screened to further identify potential missing publications.

### Study selection

Two independent authors screened the searched relevant studies based on each record’s title or abstract. Full texts were further read after the initial screening process according to the eligibility criteria. Disagreements were resolved by discussion or by a third author by tracing the original article.

### Inclusion and exclusion criteria

Studies were consider eligible if they satisfied the following criteria: (1) provided the association between the PLR level and AA; (2) the mean concentration and its standard difference (SD) of the PLR level could be obtained from original studies for both AA patients and controls (healthy controls, healthy pregnant women, outpatient clinic patients without AA, and patients with negative appendectomy) or could be calculated using the indirect methods [31, 32]; (3) provided data for the estimation of the standardized mean difference (SMD) and 95% confidence limit (CI) for the concentrations of PLR level. The exclusion criteria included: (1) reviews, comments, or meta-analysis without original data for meta-analysis; (2) studies without adequate data for abstraction; and (3) irrelevant or duplicated publications.

### Data extraction and risk of bias assessment

Pairs of independent authors extracted data from the published articles using a predefined data abstraction form. Variables concerning the study design, population characteristics and laboratory index were investigated including first author, publication year, study country and design, sample size of the study, sex percent, mean age of the participants, the blood index studied, and the type of appendicitis.

### Study quality assessment

The quality of each included study was evaluated using the Newcastle–Ottawa Quality Assessment Scale (NOS) [33]. Three domains of this scale regarding study selection, comparability, and outcome were scored with a total of nine points for observational studies. Studies obtaining a score of 7 to 9, 3 to 6, and 0 to 3 points were judged as high, moderate, and low quality, respectively [34].

### Statistical analysis

All analyses were performed using the STATA software (version 12.0; Stata Corporation). Data were collected as means ± standard deviation (SD) to estimate the pooled effect estimates. Meta-analysis was carried out using standardized mean difference (SMD) and 95% confidence limit (CI) for assessing the association between the PLR levels and AA. The random effects model, a most common and conservative approach to combine study estimates, was applied for all meta-analyses considering the between-study difference [35]. To test the stability of the meta-analysis results, we also performed the “leave-one-out” sensitivity analyses by omitting one study at each time and examining the influence of each individual study on the summary effect estimate.

### Heterogeneity assessment

Cochran’s Q test and I^2^ statistic were evaluated to assess the inter-study heterogeneity. I^2^ of 0–25%, 26–50%, 51–75% and 76–100% indicates insignificant heterogeneity, low heterogeneity, moderate heterogeneity, and high heterogeneity, respectively [36]. To further investigate the potential source of inter-study heterogeneity, we also conducted subgroup analyses based on different available variables, including sample size (≥500 vs. <500), study continent (Europe vs. Asia), patient age (adult <30 years vs. ≥30 years vs. children), appendicitis type (perforated AA vs. non-perforated AA vs. AA during pregnancy), NOS score (high quality vs. fair or low quality) and controls (healthy controls vs. healthy pregnant women vs. outpatient clinic patients without AA vs. patients with negative appendectomy).

### Publication bias assessment

Publication bias was assessed by visual inspection of the funnel plot symmetry and quantitatively by Egger’s and Begg’s tests, with a P value less than 0.05 indicating significant publication bias [37]. When publication bias existed, the Duvall and Tweedle trim-and-fill analysis would be performed to test the effects of missed study on overall effect estimates [38].

## Results

### Literature search

**Fig 1:**
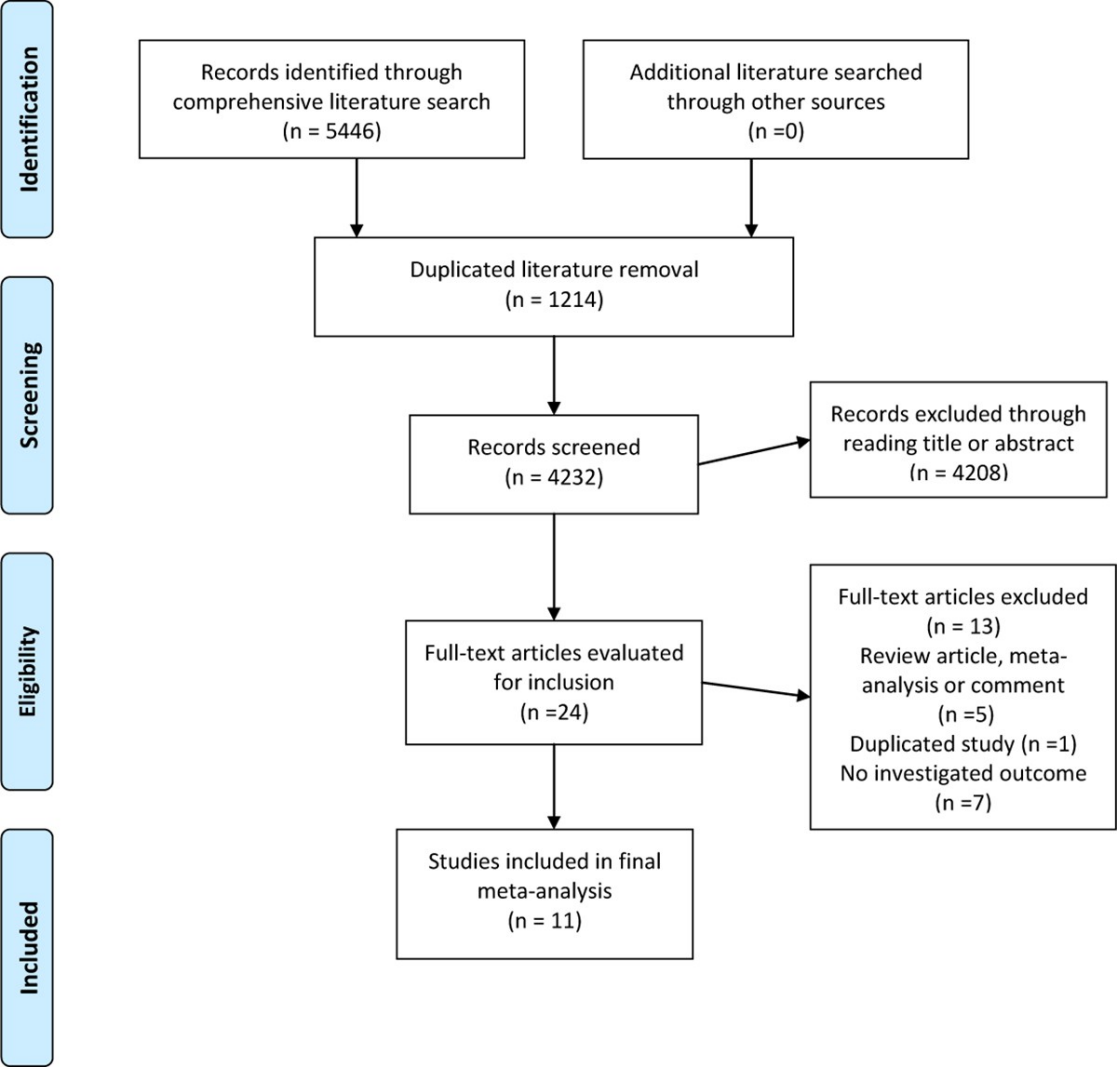
Flow diagram of the literature search and screening process.

Our initial literature search through databases identified a total of 5446 records. After removing 1214 duplicates, the remaining 4232 records were reviewed based on the title or abstract reading by two independent authors. During this stage, 4208 irrelevant studies were excluded. Twenty-four articles were retrieved and reviewed based on full text. Finally, 11 articles met the inclusion criteria and were included in our meta-analysis [26–28, 39–46]. **Fig 1** presenting a flow diagram depicts the results of the study selection process for this meta-analysis.

### Study characteristics

The baseline characteristics of each included study are shown in **Table 1**. A total of 11 studies were included in our meta-analysis with their sample size ranging from 63 to 650 subjects. The included studies were published between 2015 and 2019. Mean age of the participants ranged between 9.34 and 40 years. Two studies involved participants of pregnant women. Ten of the 11 studies reported adult patients while one included pediatric patients [28]. Five studies extracted effect estimates using indirect methods proposed by Wan et al. [26,27,40,41,45]. Most of the included studies were conducted in Europe and Asia. All of the studies used a retrospective case-control design. Based on the NOS scoring system, 8 studies ranked as high-quality (NOS score≥7) and the others as were classified as moderate in quality assessment (NOS score<7) (**S2 Table**).

**Table 1:** Characteristics of included studies.

Study	Year	Country	Sample size	Case no.	Controls and no.	Sex (male)/female %	Age(mean), years	Biomarker studied	Appendicitis type
Pehlivanli F	2019	Turkey	558	458/14	Negative appendectomy; 86	Males 55.2%; Females 44.8%	34.24	WBC, PLT, MPV, NLR, PLR	Acute appendicitis, perforated appendicitis
Yazar FM	2018	Turkey	640	511/54	Negative appendectomy; 75	Positive appendectomy (Males 54.7%; Females 45.3%); Negative appendectomy (Males 45.3%; Females 54.7%)	Positve appendectomy 39.23: Negative appendectomy 35.27	WBC, CRP, NLR, PLR	Acute appendicitis
Cinar H	2018	Turkey	94	40/7	Healthy pregnant women under routine pregnancy follow-up at the obstetrics clinics; 47	Females 100.0%	Group A 27; Group B 25.14; Control group 29.74	WBC, MPV, NLR, PLR	Acute focal appendicitis, acute suppurated appendicitis, acute perforated appendicitis, and acute gangrenous appendicitis
Nazik S	2017	Turkey	63	30	Healthy control subjects; 33	Males 65.08%; Females 34.92%	9.34	ESR,CRP, WBC, MPV, NLR, PLR, Ischemia-modified albumin	Acute appendicitis and perforated appendicitis
Kahramanca Ş	2017	Turkey	569	475	Negative appendectomy; 94	Males 55.94%; Females 44.05%	40	PLR	Acute appendicitis
Mehmet Ü	2017	Turkey	569	455	Patients with a normal appendix having other complaints in ED excluding abdominal pain; 114	Males 55.36%; Females 44.64%	31.97	WBC, PLT, Neutrophil, Lymphocyte, NLR, PLR, PDW	Acute appendicitis
Toktas O	2017	Turkey	60	30	Healthy control subjects; 30	Males 71.67%; Females 28.33%	28.5	Platelet, MPV, RDW, Neutrophil, Lymphocyte, NLR, PLR, Leukocyte	Acute appendicitis
Shin DH	2017	Korea	650	615	Negative appendectomy; 35	Males 51.2%; female 48.8%	33	WBC, NLR, LMR, PLR, DNI, CRP	Non-complicated appendicitis, complicated appendicitis
Ulukent SC	2016	Turkey	191	97	Healthy control subjects in outpatient clinics; 94	Appendicitis: males 59.8%; Females 40.2%;Control: Males 63.8%; Females 36.2%	Appendicitis 39; Control 34	Leukocyte count, neutrophil percentage, NLR, PLR, MPV, RDW, PDW and CRP	Acute appendicitis
Acar E	2016	Turkey	476	215/200	Patients without any complaints at the outpatient clinics; 61	Male 53.5%; female 46.5%	31.6	WBC, RDW, MPV, neutrophil, lymphocyte, NLR and PLR.	Acute appendicitis
Yazar FM	2015	Turkey	122	28/35	Healthy pregnant control; 29/30	Females 100.0%	Appendectomy group 26.93; Healthy pregnant control group 29.62	WBC, CRP, NLR, PLR	Acute appendicitis

Abbreviations: CRP: C-reactive protein; DNI: Delta neutrophil index; ED, emergency department; ESR: Erythrocyte sedimentation rate; MPV: Mean platelet volume; NLR: Neutrophil to lymphocyte ratio; PDW: Platelet distribution width; PLT: Platelet; PLR: Platelet to lymphocyte ratio; RDW: Red cell distribution of width; WBC: White blood cell.

### The association between PLR level and AA

**Fig 2:**
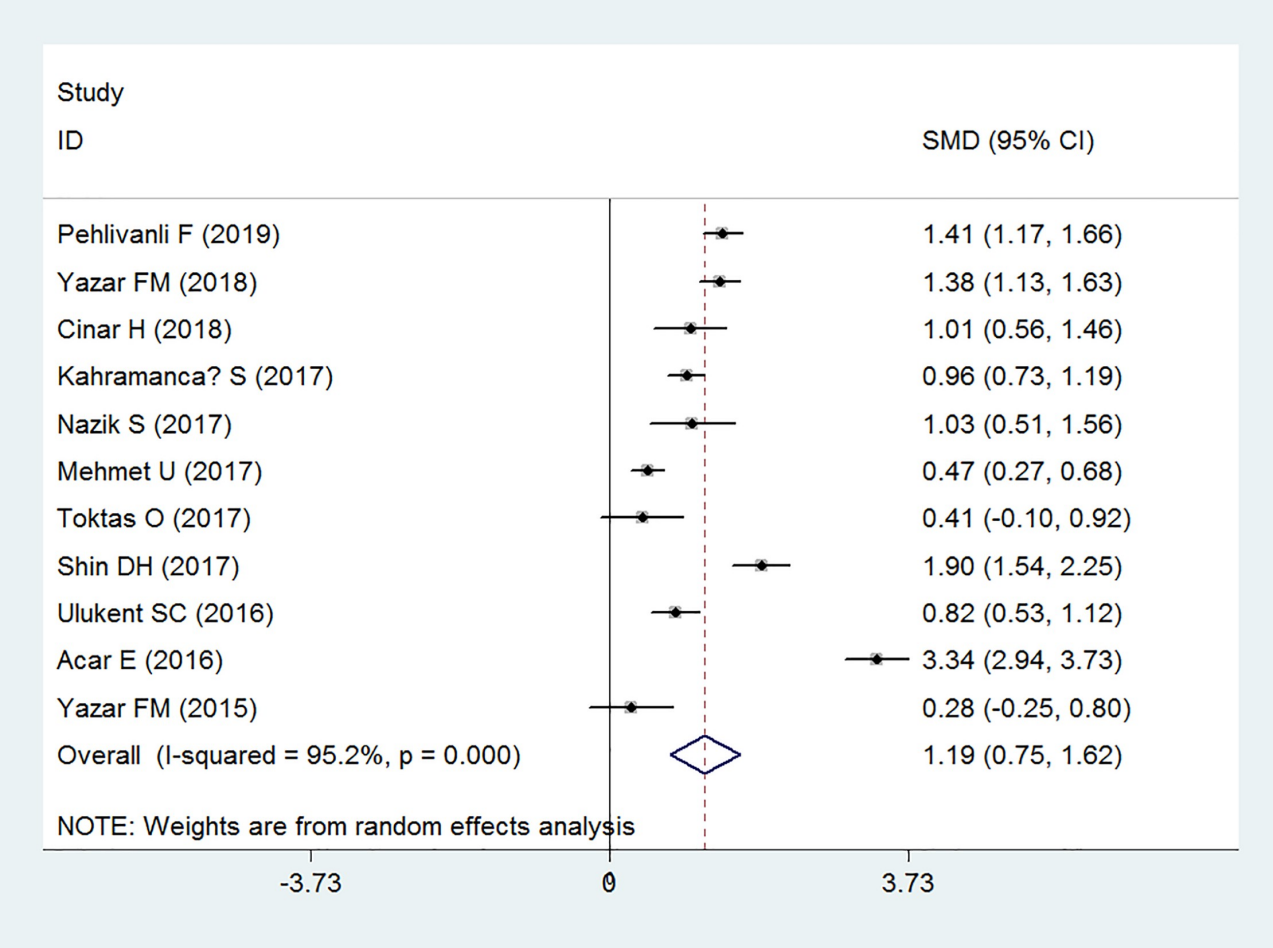
Forest plot showing the relationship between platelet to lymphocyte ratio and acute appendicitis.

Eleven case-control studies involving 3006 cases and 698 controls examined the association between PLR level and AA. Meta-analysis found that PLR levels were significantly higher in patients with AA (SMD: 1.19, 95% CI: 0.75 to 1.62, P<0.001) than that of the controls, with high inter-study heterogeneity (I^2^ = 95.2%) (**Fig 2**).

### Subgroup analysis and sensitivity analysis

We conducted several preplanned subgroup analyses and sensitivity analyses by several variables. Analyses stratified by study sample size revealed that PLR level was significantly related to AA for studies with both small sample size (<500) (SMD: 1.15; 95% CI: 0.22 to 2.08; P  = 0.015) and large sample size (≥500) (SMD: 1.21; 95% CI: 0.76 to 1.67; P <0.001). When we stratified studies by study region, a statistically significant relationship was noted for studies conducted in Europe (SMD: 1.12; 95% CI: 0.66 to 1.57; P <0.001) and in Asia (SMD: 1.90; 95% CI: 1.54 to 2.25; P = 0.003). The associations were also significant in studies with both adult patient age ≥30 years (SMD: 1.46; 95% CI: 0.89 to 2.02; P  = 0.004), adult patient age <30 years (SMD: 0.58; 95% CI: 0.12 to 1.04; P  = 0.472) and children. Sensitivity analyses revealed significant associations when involving only non-perforated AA (SMD: 1.23; 95% CI: 0.88 to 1.59; P<0.001) or AA during pregnancy (SMD: 0.70; 95% CI: 0.36 to 1.04; P = 0.037), but not perforated AA (SMD: 2.28; 95% CI: -1.72 to 6.28; P = 0.263). The PLR level was not found significantly different among complicated AA compared to that in non-complicated AA. According to the analysis by study quality, a higher PLR level was found in studies with both high quality (NOS score≥7) (SMD: 0.95; 95% CI: 0.57 to 1.32; P <0.001) and moderate or low quality (SMD: 1.84; 95% CI: 0.56 to 3.11; P  = 0.005). Based on the analyses by control subjects, significant associations were found among healthy control subjects (SMD: 0.77; 95% CI: 0.46 to 1.07; P <0.001) and control subjects with negative appendectomy (SMD: 1.40; 95% CI: 1.05 to 1.74; P <0.001). The detailed results of subgroup analyses for associations between PLR levels and AA are presented in **Table 2**.

**Table 2:** Subgroup analyses results for association between serum levels of platelet to lymphocyte ratio and acute appendicitis.

Variable	SMD	95%CI	Degree heterogeneity (I^2^ statistics; %)	*P* value	No. of included Studies	*P* for interaction
Total	1.19	0.75 to 1.62	95.2	<0.001	11	NA
Sample size						0.149
<500	1.15	0.22 to 2.08	96.4	0.015	6	
≥500	1.21	0.76 to 1.67	94.0	<0.001	5	
Study region						<0.001
Europe	1.12	0.66 to 1.57	95.2	<0.001	10	
Asia	1.90	1.54 to 2.25	-	0.003	1	
Age (Mean/median)						0.001
Adult <30 years	0.58	0.12 to 1.04	61.7	0.472	3	
Adult ≥30 years	1.46	0.89 to 2.02	96.8	0.004	7	
Children	1.03	0.51 to 1.56	-	-	1	
Appendicitis type						<0.001
Perforated AA	2.28	-1.72 to 6.28	98.9	0.263	2	
Non-perforated AA	1.23	0.88 to 1.59	75.6	<0.001	2	
AA during pregnancy	0.70	0.36 to 1.04	77.1	0.037	2	
Study quality						<0.001
NOS score≥7	0.95	0.57 to 1.32	90.3	<0.001	8	
NOS score <7	1.84	0.56 to 3.11	98.0	0.005	3	
Control subject						<0.001
Healthy subjects	0.77	0.46 to 1.07	77.1	<0.001	3	
Negative appendectomy	1.40	1.05 to 1.74	85.2	<0.001	4	
Healthy pregnant women	0.66	-0.06 to 1.37	33.3	0.074	2	
Non-AA outpatients or ED patients	1.90	-0.91 to 4.71	99.4	0.185	2	

Abbreviations: AA, acute appendicitis; CI, confidence interval; ED, emergency department; NA, not available; SMD, standardized mean difference.

**Fig 3:**
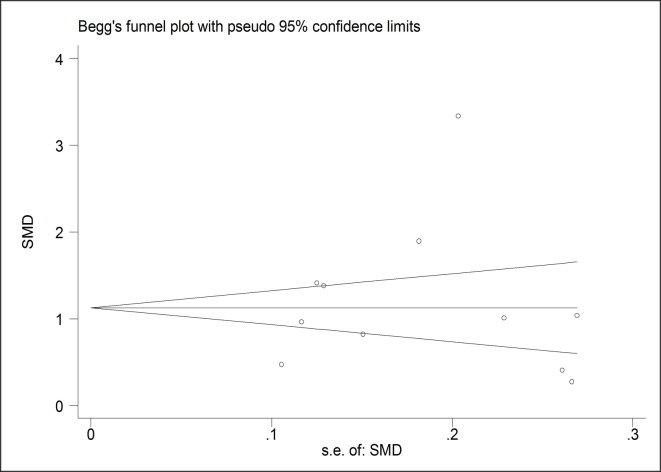
A funnel plot analysis of publication bias for association between platelet to lymphocyte ratio and acute appendicitis.

**Fig 4:**
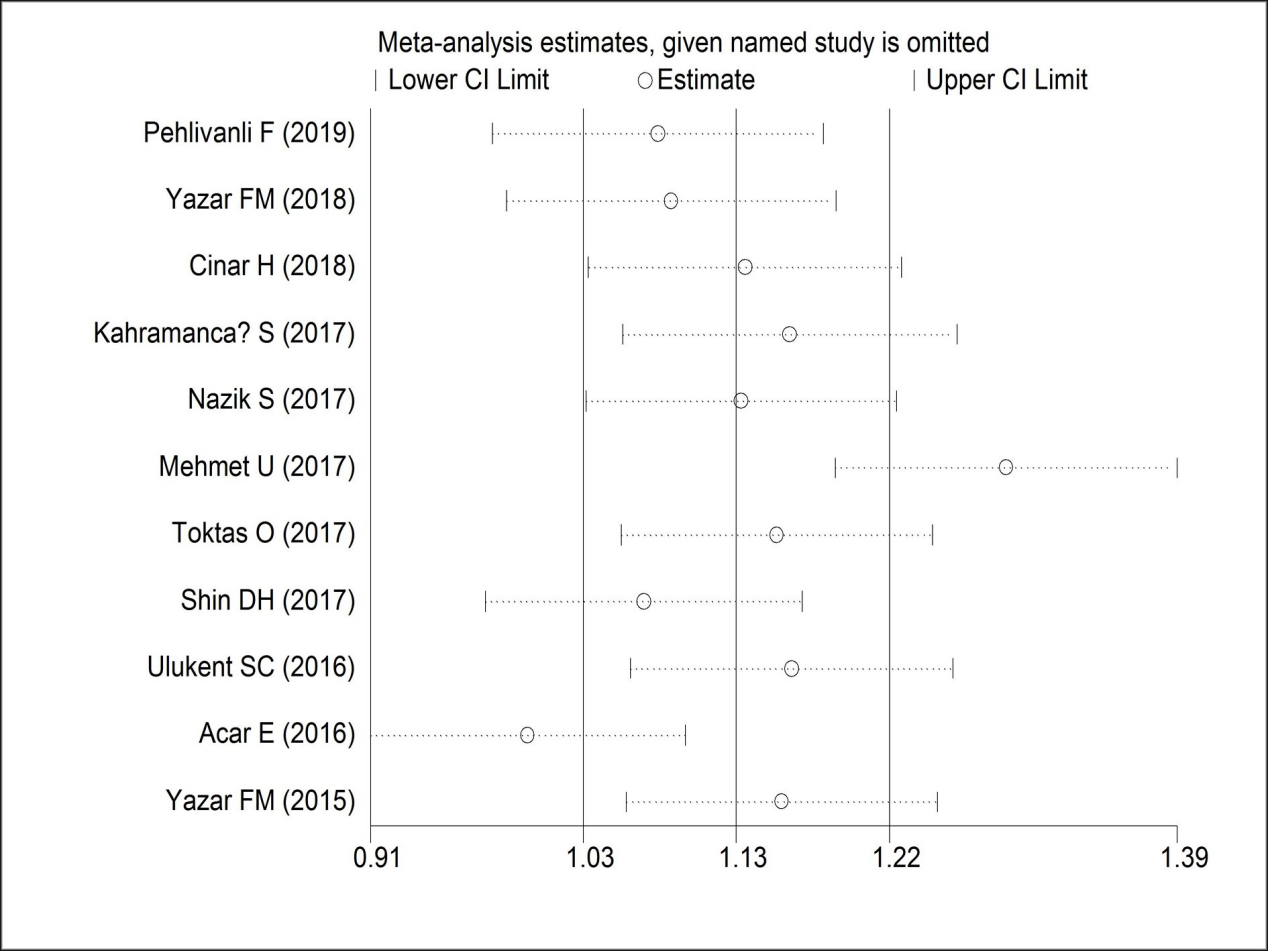
Sensitivity analysis of effect for association between platelet to lymphocyte ratio and acute appendicitis.

A funnel plot was generated to investigate the potential publication bias of the 11 included studies. Both Egger’s (P = 0.573) and Begg’s (P = 1.000) indicated that no significant publication bias existed in these studies (**Fig 3**). In addition, no filled studies were input when trim and fill method was applied, yielding the same adjusted summary SMD with the original analysis, further indicating the robustness of the finding. The “leave-one-out” sensitivity analyses by omitting one study at each time and examining the influence of each individual study also confirmed that the results were stable (**Fig 4**).

## Discussion

### Principal findings

To the best of our knowledge, this meta-analysis is the first attempt to demonstrate the association between the PLR level and AA. A comprehensive literature search of all observational studies assessing this relationship was performed. The findings indicate that the PLR level is significantly higher in AA individuals compared to the non-AA ones which is in line with previous reports [40, 42, 43]. Moreover, though the results of most subgroups are consistent with the main analysis, findings did not suggest that the PLR levels was significantly correlated with perforated AA, probably because of low statistical power with few included studies. Therefore, further well-designed prospective studies should be advocated to further confirm these relationships.

### Potential mechanisms

Numerous reports have revealed that systemic inflammatory response can induce neutrophilia and lymphocytopenia [47, 48], leading to an increase in some inflammatory indices such as PLR and neutrophil to lymphocyte ratio (NLR), some of the inflammatory biomarkers in AA [49–56]. Smith et.al found that some processes of inflammatory response were influenced by the changes in platelet markers [57]. It has been shown that cytokines such as interleukin (IL)-1 and IL-6 may affect the change of mean platelet volume (MPV), which is regarded as a marker of platelet activation inflammation [58, 59]. Studies also found that platelets can serve as key coordinators involved in several inflammatory processes [60, 61] and in the occurrence and development of several inflammatory diseases including inflammatory bowel disease, solid cancers, psoriasis multiple sclerosis and bronchial asthma [62–64]. However, few studies and meta-analyses have demonstrated the association between PLR and AA.

### Clinical relevance and implications

This study has important clinical relevance and implications. As a promising diagnostic biomarker, PLR has great potential to help in the diagnosis and decision-making of suspected appendicitis in selected populations and conditions. We found that the diagnostic implication of PLR may play an important role for participants whose age ≥30 years, pregnant women, and AA without progression to perforation. For areas with limited medical resources, due to the limited access to CT or MRI scan at the emergency department, PLR can be used as an effective rapid alternative to auxiliary diagnosis. Our study shows that PLR levels in pregnant women with appendicitis are significantly higher than those without appendicitis, which indicates that the measurement of PLR level provides an important, safe, rapid and radiation-free auxiliary diagnosis method for pregnant women suspected of appendicitis. PLR also has potential value in differentiating perforated appendicitis from nonperforated appendicitis, although large prospective studies are needed further confirmation.

The PLR, like many blood inflammatory indicators, are commonly used, non-invasive and cost-effective blood biomarkers, which can be easily available in the emergency department, even in small hospitals. However, the value of the PLR in AA has only been examined in few studies. A study conducted by Celik et al. [65] showed that the cutoff value of 284 for PLR with a sensitivity of 42% and a specificity of 86% was found to be the best predictive value for the diagnosis of complicated AA. Another study by Mehmet et al. [42] revealed that a higher PLR level was detected in patients with perforated appendicitis than that in normal controls. Pehlivanli et al. [27] assessed 558 patients who underwent appendectomy and found that the PLR appeared to be significantly valuable in the differentiation of normal appendix from AA and in the differentiation of AA from perforated appendicitis.

In this study, the reasons to select SMD as the statistical parameter for this kind of meta-analysis were explained as follows. First, the current literature search did not yield sufficient eligible studies for diagnostic meta-analysis combining the sensitivity and sensitivity of PLR as effect estimates. Instead, 11 studies could be identified providing means and SDs of PLR levels for both AA patients and controls, which could be used as an alternative effect estimate to conduct a meta-analysis. Thus, SMD is a suitable substitute as a statistical parameter to demonstrate the associations between PLR levels (a continuous measure outcome) and AA. Second, a research article published in 2019 [66] has demonstrated that SMD is a commonly used effect estimate to measure continuous outcomes using different scales or units (e.g., blood inflammatory biomarkers with different units). These effect estimates should be standardized before pooling in a meta-analysis. One of the frequently used methods of standardization includes the use of SMD. In our meta-analysis, we also used SMD because units of PLR level in the included studies were different. Finally, the result of our study can be well interpreted. We found that the level of PLR in the AA group was significantly higher than that in the control group by 1.19 standard deviations of PLA levels (SMD: 1.19, 95% CI: 0.75 to 1.62, P<0.001), which indicates that PLR is a promising blood biomarker that can potentially predict AA in patients with clinical suspicion of appendicitis.

Considerable inter-study heterogeneity was detected in our meta-analysis, though we conducted several subgroup analyses. The results of subgroup analyses for relationship between the PLR level and AA indicated that the heterogeneity could partly attribute to variables regarding differences in patient age, appendicitis type and control subjects. However, these variables could not account for all of the heterogeneity. Perhaps further larger well-designed prospective cohort studies or clinical trials are warranted in the future to better demonstrate the relationship between the PLR level and AA.

### Strengths and limitations

The present meta-analysis has several advantages. Firstly, this is the first meta-analysis of the relationship between PLR level and AA. From the pooled analysis, we found that patients with AA had significant higher PLR level than the non-AA controls. Moreover, multiple analyses including the “leave-one-out” sensitivity analysis, trim and fill method and subgroup analyses confirmed this association was stable. In addition, the results of subgroup analyses were mostly consistent with those of the main analysis, which also indicated our findings were convincing. Secondly, random-effects model, a more conservative approach, was used when we pooled effect estimates, making the results of our meta-analysis more accurate. Thirdly, publication date and language limits were not restricted for literature search, making the results less possible to leading to publication bias.

This meta-analysis should still be interpreted with caution due to the limitations in several aspects. First, despite that all studies used blood sample analyzer to evaluate PLR levels, the sample collection time and reference values were varied, which can lead to heterogeneous results. Second, besides the lack of diagnostic accuracy studies, the results of this meta-analysis were mainly based on observational studies, mostly case-control studies, which is a limitation of critical importance for demonstrating a causal relationship between PLR level and AA. However, observational study results may also provide significant preliminary data to justify further larger trials and well-designed prospective cohort studies. Third, there seem to be patients mostly from one continent of Europe. The variations on the ethnicity of the population are still under investigation. Because limited number of studies is involved, multi-ethnic cohort studies are warranted to confirm our findings. Fourth, it is known that systemic inflammatory response might induce an increase in blood PLR. It is quite obvious that AA can also lead to the PLR increase. Diagnosis of AA can be challenging due to the fact that a variety of acute gastrointestinal and gynaecologic diseases can mimic the symptoms of AA. Therefore, more high quality evidence should be accumulated before definite conclusions could be drawn. Last but not least, as we extracted estimates using indirect methods in 5 studies, this method might have led to overestimation or underestimation of the sample of SD and thereby causing inaccuracy of the effect estimates.

In conclusion, this meta-analysis shows a significant increase in PLR level in patients with AA, indicating that PLR is a promising biomarker for AA. PLR provides a convenient option for emergency department to quickly screen for clinically or radiologically confirmed AA awaiting appendectomy, especially for pregnant women suspected of having AA. For areas with limited medical resources, due to the limited access to CT or MRI scans in emergency department, PLR can be used as an effective and rapid alternative to auxiliary diagnosis. However, more high-quality evidence is needed to further confirm the diagnostic accuracy of PLR for AA, and whether PLR combined with other blood biomarkers will yield better predictive value needs to be further studied.

## Supporting information

S1 File Appendix A. Supplementary search strategies.
(DOCX)

S1 Table The PRISMA checklist. (DOC)

S2 Table Quality assessment of included studies based on Newcastle-Ottawa Scale. (DOCX)
