# Supplementary material for: Is the platelet to lymphocyte ratio a promising biomarker to distinguish acute appendicitis? Evidence from a systematic review with meta-analysis
Source: PLoS One. 2020 May 22;15(5):e0233470. doi: 10.1371/journal.pone.0233470 (PMC7244160; doi:10.1371/journal.pone.0233470)
Supplement: S1 File — Supplementary search strategies. (DOCX) [file pone.0233470.s001.docx]

**S1 File**

**Supplementary Search Strategies**

**Pubmed**

| 1. "Appendicitis"[Mesh] |
| --- |
| 1. "Appendectomy"[Mesh] |
| 1. (appendi* OR appendec*) [Title/Abstract] |
| 1. 1-3/OR |
| 1. "Lymphocyte Count"[Mesh] |
| 1. "Lymphocytes"[Mesh] |
| 1. "Lymphocyte Count"[Mesh] |
| 1. (“platelet to lymphocyte ratio” OR “platelet lymphocyte ratio” OR “platelet-to-lymphocyte ratio” OR PLR) [Title/Abstract] |
| 1. 5-8/OR |
| 1. 4 AND 9 |

**Embase**

| 1. 'appendicitis'/exp |
| --- |
| 1. 'appendectomy'/exp |
| 1. (appendi* OR appendec*): ab.ti |
| 1. 1-3/OR |
| 1. 'lymphocyte count'/exp |
| 1. 'lymphocyte'/exp |
| 1. 'lymphocyte count'/exp |
| 1. (‘platelet to lymphocyte ratio’ OR ‘platelet lymphocyte ratio’ OR PLR): ab.ti |
| 1. 5-8/OR |
| 1. 4 AND 9 |

**Cochrane Library Central Register of Controlled Trials**

#1 MeSH descriptor: [Appendicitis] explode all trees

#2 MeSH descriptor: [Appendectomy] explode all trees

#3 (appendi* OR appendec*):ti,ab,kw (Word variations have been searched)

#4 #1 or #2 or #3

#5 MeSH descriptor: [Lymphocyte Count] explode all trees

#6 MeSH descriptor: [Lymphocytes] explode all trees

#7 MeSH descriptor: [Lymphocyte Count] explode all trees

#8 (appendi* OR appendec*“platelet to lymphocyte ratio” OR “platelet lymphocyte ratio” OR “platelet-to-lymphocyte ratio” OR PLR):ti,ab,kw (Word variations have been searched)

#9 #5 or #6 or #7 or #8

#10 #4 and #9
