# Supplementary material for: Is the platelet to lymphocyte ratio a promising biomarker to distinguish acute appendicitis? Evidence from a systematic review with meta-analysis
Source: PLoS One. 2020 May 22;15(5):e0233470. doi: 10.1371/journal.pone.0233470 (PMC7244160; doi:10.1371/journal.pone.0233470)
Supplement: S2 Table — (DOCX) [file pone.0233470.s003.docx]

**S2 Table. Quality assessment of included studies based on Newcastle-Ottawa Scale**

| **Study** | **Year** | **Adequate definition of cases** | **Representativeness of cases** | **Selection of control subjects** | **Definition of control subjects** | **Control for important factor or additional factor** | **Exposure assessment** | **Same method of ascertainment for all subjects** | **Non-response rate** | **Score** |
| --- | --- | --- | --- | --- | --- | --- | --- | --- | --- | --- |
| Yazar | 2018 | * | * |  | * |  | * | * | * | 6 |
| Cinar | 2018 | * | * |  | * | * | * | * | * | 7 |
| Pehlivanli | 2019 | * | * |  | * | * | * | * | * | 7 |
| Nazik | 2017 | * | * |  | * | * | * | * | * | 7 |
| [Kahramanca](http://xueshu.baidu.com/s?wd=author%3A%28Kahramanca%20S%29%20&tn=SE_baiduxueshulib_9r82kicg&ie=utf-8&sc_as_para=sc_lib%3Azzu&sc_from=zzu&sc_f_para=sc_hilight%3Dperson) | 2017 | * | * |  | * | * | * | * | * | 7 |
| Mehmet | 2017 | * | * |  | * | ** | * | * | * | 8 |
| Toktas | 2017 | * | * |  | * | ** | * | * | * | 8 |
| Yazar | 2015 | * | * |  | * | * | * | * | * | 7 |
| Ulukent | 2016 | * | * |  | * | * | * | * |  | 6 |
| Acar | 2016 | * | * |  | * | * | * | * |  | 6 |
| Shin | 2017 | * | * |  | * | ** | * | * |  | 7 |
